# Widespread hnRNP K Mislocalisation Suggests Differential Neuronal Vulnerability in the Neurodegenerative and Ageing Human Brain

**DOI:** 10.1111/nan.70072

**Published:** 2026-04-08

**Authors:** Xinwa Jiang, Christina E. Toomey, Tammaryn Lashley, Ariana Gatt

**Affiliations:** ^1^ Department of Neurodegenerative Disease, Queen Square Institute of Neurology University College London London UK

**Keywords:** frontotemporal dementia, frontotemporal lobar degeneration, heterogeneous nuclear ribonucleoproteins, hnRNP K

## Abstract

Heterogeneous nuclear ribonucleoprotein K (hnRNP K) is a widely distributed RNA‐binding protein in the human brain, playing a crucial role in post‐transcriptional regulation, including mRNA metabolism and neuroplasticity. We have previously identified an increase in neuronal hnRNP K mislocalisation in cases of frontotemporal lobar degeneration (FTLD) compared to controls, where loss of nuclear hnRNP K was linked to alternative splicing events. However, the broader distribution of hnRNP K mislocalisation across different brain regions, other diseases and its pathological significance remains unclear. This study systematically examined hnRNP K mislocalisation across 13 brain regions from 19 cases, including different pathological subtypes of FTLD, Parkinson's disease (PD), Alzheimer's disease (AD) and age‐matched neurologically normal controls, using immunohistochemistry and quantitative image analysis. The results of the study show that hnRNP K mislocalisation is observed throughout the brain, characterised by nuclear depletion and cytoplasmic aggregation. In the cerebral cortex, mislocalisation was most pronounced in the frontal lobe and least in the occipital lobe, with significant predominance in the depth of sulci compared to gyri. Notably, the basal ganglia, thalamus, medulla and cerebellum exhibited particular vulnerability to hnRNP K pathology. In contrast, Purkinje cells within the cerebellum and CA1–CA2 pyramidal neurons within the hippocampus showed lower levels of mislocalisation. Furthermore, levels of hnRNP K mislocalisation within the putamen correlated significantly with motor symptoms, suggesting a potential link between hnRNP K pathology and motor dysfunction. These findings highlight the propensity of hnRNP K mislocalisation in neurodegenerative diseases and the aged brain and underscore the need for further investigation into its functional consequences.

## Summary

- This study provides the first systematic histological mapping of hnRNP K mislocalisation across multiple brain regions in neurodegenerative disease and ageing.
- It demonstrates widespread but regionally heterogeneous hnRNP K mislocalisation, with prominent involvement of thalamus, basal ganglia, medulla and cerebellar dentate nucleus.
- The findings identify selective neuronal vulnerability, with cortical pyramidal neurons affected, while Purkinje cells and CA1–CA2 hippocampal neurons are relatively preserved.
- We found a consistent sulcal predominance of hnRNP K mislocalisation across cortical regions
- The study reveals an association between hnRNP K mislocalisation in the putamen and dyskinesia, implicating RNA‐binding protein dysfunction in motor system pathology.

## Graphical abstract

Widespread neuronal hnRNP K mislocalisation, characterised by nuclear loss and cytoplasmic aggregation, was identified across multiple brain regions in FTLD, PD and AD. Regional vulnerability patterns and correlations with motor symptoms suggest hnRNP K pathology as a common feature of neurodegeneration with potential functional and clinical significance.

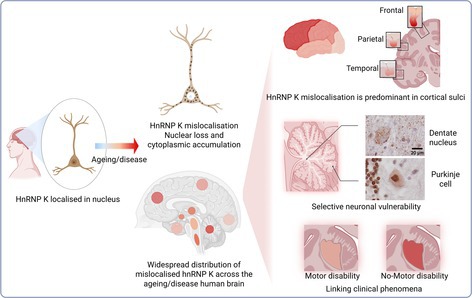

## 1 Introduction

Heterogeneous nuclear ribonucleoproteins (hnRNPs) are a group of RNA‐binding proteins (RBPs) that play crucial roles in the regulation of mRNA processing and metabolism. They function through dynamic interactions with RNA molecules and other RBPs within RNA‐protein complexes [1].

Differing from other RBPs, such as SR splicing factors and messenger RNPs (mRNPs), hnRNPs follow a unique nomenclature that extends alphabetically from A to U [1]. The dysregulation of hnRNPs has been linked to numerous diseases, including cancer and developmental disorders [2, 3, 4]. In recent years, abnormalities in hnRNPs have also been found to be associated with neurodegenerative diseases, especially frontotemporal lobar degeneration (FTLD) and amyotrophic lateral sclerosis (ALS) [5, 6, 7, 8]. The well‐characterised protein TDP‐43 (transactive response DNA‐binding protein 43) is also a member of the hnRNP family, even though it does not follow the standard nomenclature. TDP‐43 is mislocalised from the nucleus to cytoplasmic inclusions in up to 97% of ALS cases and 45% of FTLD cases [9, 10, 11], and TDP‐43 proteinopathy has therefore been identified as the most common pathology found in both ALS and FTLD. In addition to ALS and FTLD, TDP‐43 pathology is also the defining feature of LATE, an age‐related limbic‐predominant encephalopathy frequently associated with AD‐like cognitive impairment [12].

HnRNP K is among the most widely expressed RBPs in the human brain, functioning across diverse cell types to regulate gene expression and RNA metabolism. It contains six functional domains: three KH domains (KH1, KH2 and KH3), which are responsible for RNA binding; two domains associated with nuclear localisation and shuttling (NLS and KNS); and a distinctive K interactive (KI) domain. The nucleocytoplasmic shuttling domains enable transport between the nucleus and cytoplasm [13]. Additionally, the KH domains of hnRNP K can bind specific DNA sequences—KH1 and KH2 interact with the promoter's C‐rich sequence region of the oncogene c‐Myc, influencing gene transcription. They also bind to the oncogenic factor p53, activating a series of cell cycle‐ and apoptosis‐related genes in response to cellular stress [14]. While its potential pathogenic roles have traditionally been examined more thoroughly within oncological contexts, emerging studies have shed light on its indispensable roles in neurological development and maintenance, encompassing axogenesis, myelination, synaptic plasticity and the maintenance of ATP levels under cellular stress [9, 15, 16, 17, 18, 19].

Several pathogenic variants in *HNRNPK* have been linked to neurodevelopmental disorders, notably Au‐Kline syndrome [3, 4, 20]. This rare syndrome results from heterozygous loss‐of‐function or missense variants affecting hnRNP K [3, 4]. Although the mechanisms connecting *HNRNPK* variants with neurodevelopmental impairments are not fully understood, studies suggest a role in chromatin regulation, as unique DNA methylation patterns within 429 CpG sites distinguish patients from controls [4]. Dysfunction in downstream RNA processing may also play a role, as approximately 85% of pathogenic *HNRNPK* missense variants cluster in the KH domains, the primary regions for RNA recognition and binding [4].

In addition to its role in neurodevelopmental disorders, hnRNP K has been recently implicated in neurodegenerative diseases. Recent studies from our lab have shown significant mislocalisation of hnRNP K from the nucleus to the cytoplasm in the cortical regions of brains affected by FTLD [21]. Distinct neuronal cytoplasmic hnRNP K puncta observed within the grey matter do not co‐localise with established neuropathological markers such as phosphorylated TDP‐43 (pTDP43), tau or p62, marking a unique pathological signature. In a subsequent study, our group also observed mislocalisation of hnRNP K in the dentate nucleus of the cerebellum, as well as in CA4 neurons of the hippocampus in Alzheimer's disease (AD) [22]. *HNRNPK* knockdown in cellular models leads to notable transcriptional and splicing disruptions, including the abnormal inclusion of cryptic exons within transcripts [21]. Moreover, hnRNP K is mislocalised in response to osmotic stress in iPSC‐derived motor neurons [23]. These findings underscore the need for further research into the pathological functions of hnRNP K in neurodegenerative diseases.

As one of the RBPs most widely expressed in the brain, the effects of mislocalised hnRNP K are unlikely to be confined to a single brain region. In this study, we investigate whether similar levels of hnRNP K mislocalisation occur across different brain regions, whether cases with high levels of mislocalisation in the frontal cortex exhibit comparable distribution patterns throughout the rest of the brain, and whether the extent of mislocalisation correlates with the functional relevance of each affected region. Furthermore, we aimed to assess whether consistent patterns of hnRNP K mislocalisation are observed across different neurodegenerative diseases. To address these questions, we analysed 13 brain regions from 19 cases spanning various neurodegenerative diseases, including FTLD subtypes, AD, Parkinson's disease (PD) and control cases with no clinical manifestation of neurological disease, using quantitative imaging following immunohistochemical staining.

In this study, we present the first histological map of hnRNP K mislocalisation in the human neurodegenerative brain, revealing both regional and cell‐type‐specific vulnerability. Mislocalisation is most prominent in the thalamus, medulla and cerebellum. Furthermore, within the cerebral cortex, mislocalisation was most pronounced in the frontal lobe and least in the occipital lobe. Certain neuronal populations, such as Purkinje cells and CA1–2 hippocampal neurons, are resistant to hnRNP K mislocalisation, while others, including those in the dentate nucleus and CA4, show marked mislocalisation. We also identified a consistent sulcal dominance in cortical regions and a potential link between mislocalisation in the putamen and dyskinesia. These findings suggest that hnRNP K mislocalisation reflects selective vulnerability and may contribute to region‐specific pathogenesis in neurodegeneration. Collectively, these findings provide novel insights into the regional and cellular dynamics of hnRNP K mislocalisation and offer new avenues for investigating its role in the pathogenesis of neurodegenerative diseases.

## 2 Material and Methods

### 2.1 Cases

Brains were donated to the Queen Square Brain Bank for Neurological Disorders, UCL Queen Square Institute of Neurology (QSBB), with full informed consent. Clinical and demographic data for all cases used in this study were stored electronically in compliance with the 1998 Data Protection Act. Ethical approval for the study was obtained from the NHS Research Ethics Committee (NEC) and conducted in accordance with the Human Tissue Authority's (HTA) Code of Practice and Standards under licence number 12198.

Tissue samples used for staining were obtained from formalin‐fixed, paraffin‐embedded wax blocks from the following brain regions: frontal, temporal, parietal and occipital cortices; cingulate cortex; hippocampus; amygdala; basal ganglia; thalamus; midbrain; pons; medulla; and cerebellum. Cases were selected based on the degree of hnRNP K mislocalisation identified in the frontal cortex during preliminary screening. Selection was guided by both quantitative mislocalisation scoring and prior evidence of hnRNP K pathology [21, 22]. To ensure representation across the spectrum of pathologies, cases with both high and low hnRNP K mislocalisation scores were intentionally included. The cohort comprised the following: FTLD with TDP‐43 pathology type A (FTLD‐TDP A; *n* = 3), including two cases harbouring *C9orf72* repeat expansions; FTLD‐TDP type C (*n* = 2); FTLD‐tau (*n* = 4); FTLD‐FUS (*n* = 2); familial Alzheimer's disease (FAD; *n* = 1); PD (*n* = 2). These diagnostic groups were selected to enable comparison across major molecular subtypes of FTLD and related neurodegenerative disorders with distinct proteinopathies (TDP‐43, tau and FUS), as well as synucleinopathy and amyloid‐driven pathology. Inclusion of these groups allowed assessment of whether hnRNP K mislocalisation is preferentially associated with specific pathological substrates or represents a broader neurodegenerative phenomenon. Initially, three neurologically normal controls were included. Following preliminary analysis, two additional control cases were incorporated. This decision was made after early findings indicated that cases with non‐PD tremor demonstrated unexpectedly higher hnRNP K mislocalisation in the putamen, necessitating expansion of the control group to ensure appropriate comparison and to account for potential movement disorder–related confounding effects.

Together, this strategy ensured representation of a range of hnRNP K mislocalisation severities, underlying molecular pathologies and clinically relevant comparison groups, while remaining grounded in prior pathological observations. A demographic summary of all cases used in this study is shown in Table 1.

**TABLE 1:** Case demographic information.

QSBB number	Pathological diagnosis	AOO	AAD	Gender	Brain weight	Mislocalisation of hnRNP K in frontal sulcus	Motor disability
1	FTLD‐TDPA *(C9orf72)*	54	60	M	1350	1.0%	No
2	FTLD‐TDPA	66	72	M	1274	36.9%	No
3	FTLD‐TDPA *(C9orf72)*	53	63	M	955	45.4%	No
4	FTLD‐TDPC	59	73	F	936	2.0%	No
5	FTLD‐TDPC	77	80	F	1502	62.9%	Yes
6	FTLD‐Tau *(MAPT(10 + 16))*	59	66	M	1399	1.3%	No
7	FTLD‐Tau *(MAPT(k280del))*	68	74	M	1048	36.9%	No
8	FTLD‐Tau (*MAPT*)	57	63	M	1281	50.9%	No
9	FTLD‐Tau *(MAPT(R406W))*	55	66	M	1208	64.7%	No
10	FTLD‐FUS (NIFID)	69	72	F	1268	23.8%	No
11	FTLD‐FUS	44	46	M	1570	46.0%	Yes
12	FAD (*PS1*)	42	47	M	1225	17.0%	No
13	PD	60	76	M	1761	0%	No
14	PD	55	71	M	1490	30.5%	Yes
15	Control with no PD tremor	na	91	F	1130	1.2%	Yes
16	Control with no PD tremor	na	96	F	1032	4.5%	Yes
17	Control with no PD tremor	na	86	F	1234	81.5%	Yes
18	Control	na	68	F	1330	0.3%	No
19	Control	na	80	F	1242	23.1%	No

*Note:* The case demographic data for the cases used within the project are detailed in the table. The brackets in the table show the pathogenic variants for the different cases. For motor disability, ‘No’ indicates the absence of any recorded movement‐related symptom from the clinical notes for the case. From the 19 analysed cases, six cases had a recorded movement disorder: Cases 5, 15, 16, 17 had non‐PD tremor, case 14 had dyskinesia and Case 11 had facial dyskinesia.

Abbreviations: AAD, age at death; AOO, age of onset; FAD, familial Alzheimer's disease; FTLD, frontotemporal lobar degeneration; FTLD‐TDP A, frontotemporal lobar degeneration TAR DNA‐binding protein 43 subtype A; FTLD‐TDP C, frontotemporal lobar degeneration TAR DNA‐binding protein 43 subtype C; na, not applicable; PD, Parkinson's disease.

### 2.2 Immunohistochemistry for hnRNP K

Eight‐micrometre‐thick formalin‐fixed paraffin‐embedded (FFPE) sections were cut from 13 brain regions from 19 cases (Table 1). The sections were deparaffinised in xylene and rehydrated using graded alcohols. Endogenous peroxidase activity was blocked using 0.3% H_2_O_2_ in methanol for 10 min, followed by pressure cooker pretreatment for 10 min in citrate buffer, pH 6.0. Non‐specific binding was blocked using 10% dried milk/tris‐buffered saline‐Tween (TBS‐T) before incubating with an hnRNP K (mouse‐derived monoclonal; Abcam ab23644, 1:1000) primary antibody overnight at 4°C. A biotinylated anti‐mouse IgG antibody (1:200, 30 min, DAKO) was incubated with the sections at room temperature, followed by avidin‐biotin complex (30 min, Vector Laboratories). The colour was developed with di‐aminobenzidine, activated with H_2_O_2_. Sections were scanned on an Evident VS100 slide scanner.

### 2.3 Image Quantification: hnRNP K Mislocalisation

The 19 cases selected for this study (Table 1) spanned varying degrees of hnRNP K mislocalisation in the frontal cortex. Cells exhibiting hnRNP K mislocalisation, along with morphologically normal neurons, were manually counted within selected regions of interest (ROIs). The percentage of mislocalised cells to normal neurons was then used to represent the level of hnRNP K mislocalisation in each brain region. The extent of hnRNP K mislocalisation in the frontal sulcus among the 19 selected cases ranged from 0.3% to 81.5%. HnRNP K‐stained sections were analysed using QuPath to delineate ROIs, which were then transferred to ImageJ and subdivided into same size images using macros. Each image was standardised to 1000 × 1000 pixels (345.34 × 345.34 μm^2^). The number of equal‐sized images obtained per ROI depended on the size of the ROI. In order to make sure that randomly selected images within each ROI did not overlap in space, gaps were left between images, resulting in a maximum area coverage rate of 45% per ROI (Figure S1 and Table S2). Cells with mislocalised hnRNP K were manually counted. The total number of neurons within each image was also manually counted. Image analysis was conducted in a double‐blind manner, with results expressed as the percentage of hnRNP K mislocalised neurons relative to the total neurons counted.

### 2.4 Image Quantification: Soma Size Measurement

Soma size was measured in the frontal sulcus, frontal gyrus and CA4 region. ROIs were exported from QuPath and imported into ImageJ, where they were divided into uniformly sized images of 1000 × 1000 pixels. The soma size of both normal and hnRNP K‐mislocalised pyramidal cells was measured using ImageJ. Owing to the high density of normal pyramidal cells in the gyrus, only a subset was analysed. To minimise selection bias, a grid‐based random sampling method was employed, whereby cells intersecting the grid lines (5000 μm^2^ per point) were selected for measurement. The polygon selection tool in ImageJ was used to manually outline each soma, generating numerical area values. The average soma size for mislocalised and normal cells was calculated across all cells counted per ROI. To ensure that hnRNP K staining could reliably reflect soma size, haematoxylin and eosin (H&E) and crystal violet (CV) staining results from each case were included for comparison. No differences in soma size were observed between the two staining methods.

## 3 Results

### 3.1 Baseline Characterisation of hnRNP K Immunostaining in Human Brain

In baseline conditions, hnRNP K staining is predominantly nuclear, reflecting its principal localisation and function. As hnRNP K is capable of shuttling between the nucleus and the cytoplasm, a lower level of the protein will also be present in the cytoplasm, reflected as a fainter stain in relation to nuclear immunostaining. Normal hnRNP K staining does not exhibit cytoplasmic aggregation.

**FIGURE 1:**
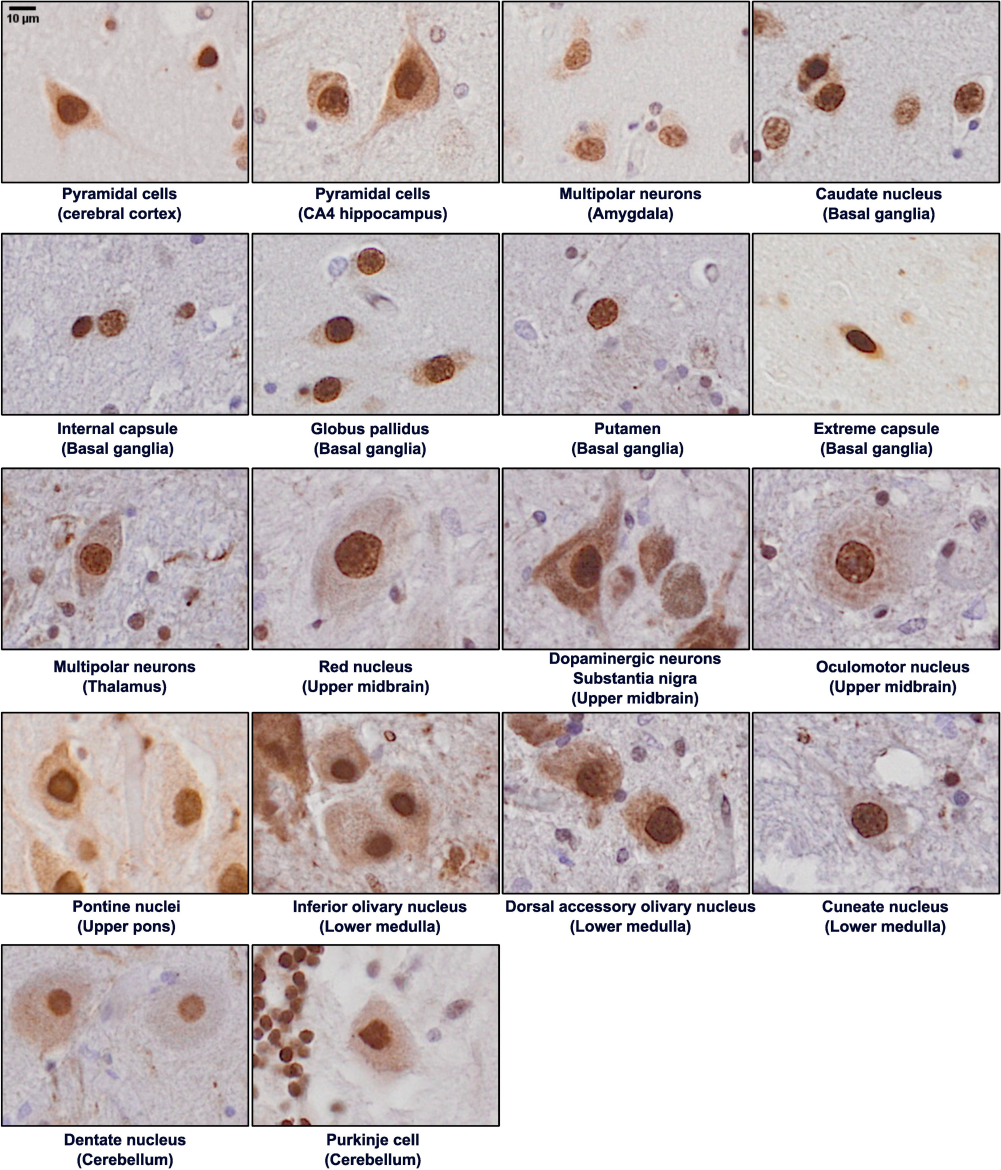
Normal hnRNP K staining and cell morphology in different neuronal populations. Pyramidal neurons exhibit the retention of hnRNP K in the nucleus across most brain regions. To illustrate normal hnRNP K staining patterns in pyramidal neurons, under normal physiological conditions, hnRNP K has a strong nuclear and minimal cytoplasmic presence within the neurons, which can be observed across all brain regions and cell types. Scale bar represents 10 μm in all images.

**FIGURE 2:**
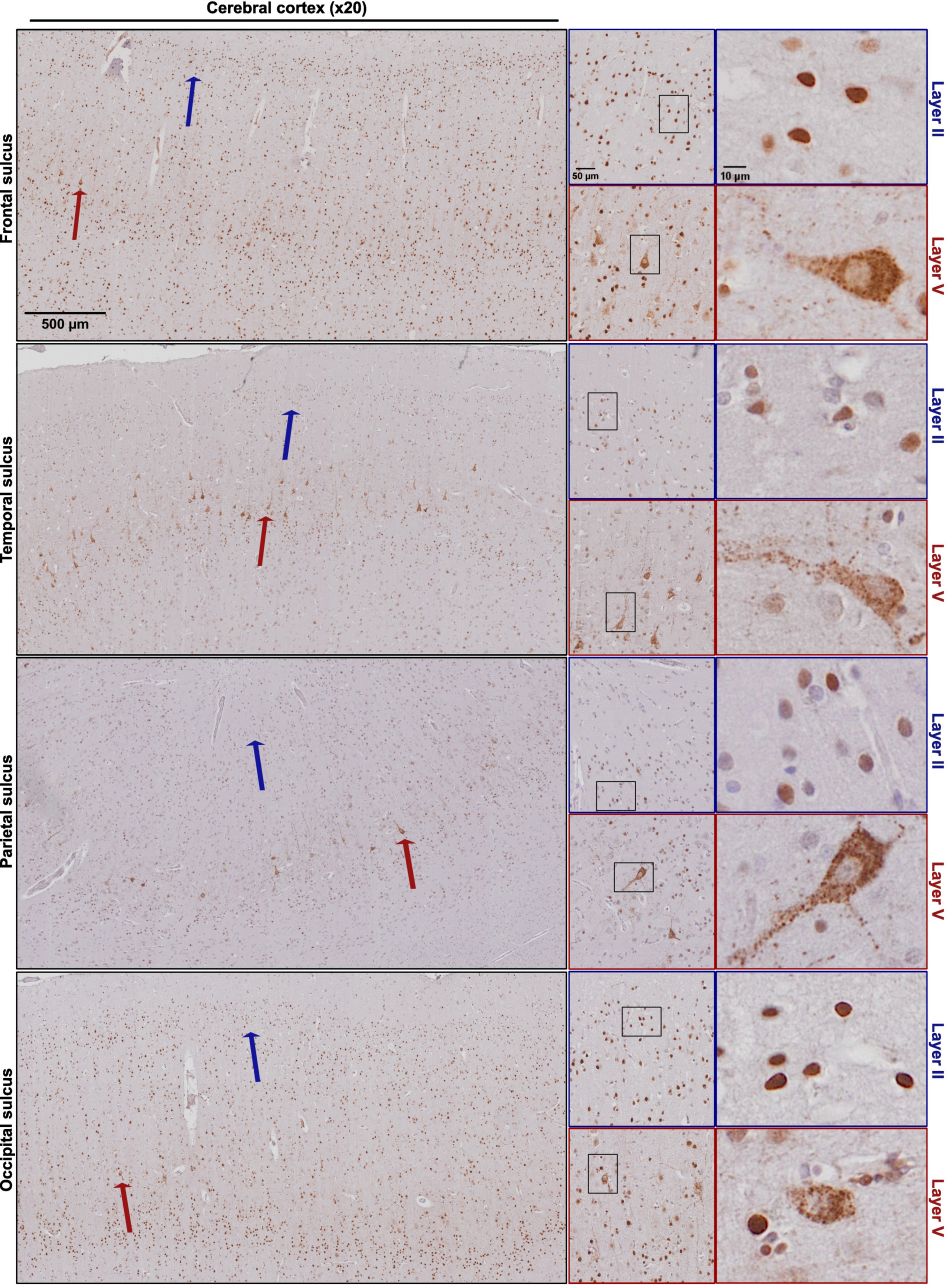
Representative images of hnRNP K mislocalisation in different cortical regions. The panels on the left show low power hnRNP K immunohistochemical images from the frontal, temporal, parietal and occipital cortices. The blue arrows highlight layer II of the cortex, and the red arrows highlight layer V. Middle and right panels show higher power images to demonstrate the nuclear retention of hnRNP K in the layer II neurons and hnRNP K mislocalisation in layer V pyramidal neurons. Scale bars are as shown on the representative images.

In conditions of hnRNP K mislocalisation, nuclear staining is depleted, and distinct hnRNP K puncta appear in the cytoplasm (Figure 2). It is important to carefully distinguish true hnRNP K punctate staining from other sources of intracellular pigmentation, such as the intrinsic pigmentation of melanin in dopaminergic neurons and Nissl body staining in oculomotor neurons, both of which can be easily misinterpreted as hnRNP K aggregates (Figure 1). In H&E staining, melanin appears yellowish‐brown, while in immunohistochemical staining, it presents as darker and denser compared to the hnRNP K signal. In oculomotor neurons, Nissl body staining tends to be more homogeneous and exhibits blurred edges, in contrast to the discrete, punctate appearance of hnRNP K aggregates.

### 3.2 Neuronal Vulnerability to hnRNP K Mislocalisation Across Cortical Regions

We determined the spatial distribution of hnRNP K mislocalisation within the cerebral cortex by examining immunohistochemical staining patterns across four cortical regions: frontal, temporal, parietal and occipital cortices (Figure 2). In all regions examined, hnRNP K mislocalisation was limited to the pyramidal neurons of cortical layers III and V. These neurons exhibited characteristic nuclear depletion of hnRNP K, accompanied by punctate cytoplasmic aggregates. In contrast, cortical neurons in layer II retained normal nuclear localisation of hnRNP K, indicating a selective vulnerability of deeper‐layer pyramidal neurons to hnRNP K mislocalisation (Figure 2) as had previously been observed only in the frontal cortex [21]. This layer‐specific distribution suggests that certain neuronal subtypes, particularly those with larger somas and long‐range projections, may be more susceptible to hnRNP K mislocalisation.

### 3.3 Regional Variation in hnRNP K Mislocalisation Between Gyri and Sulci

**FIGURE 3:**
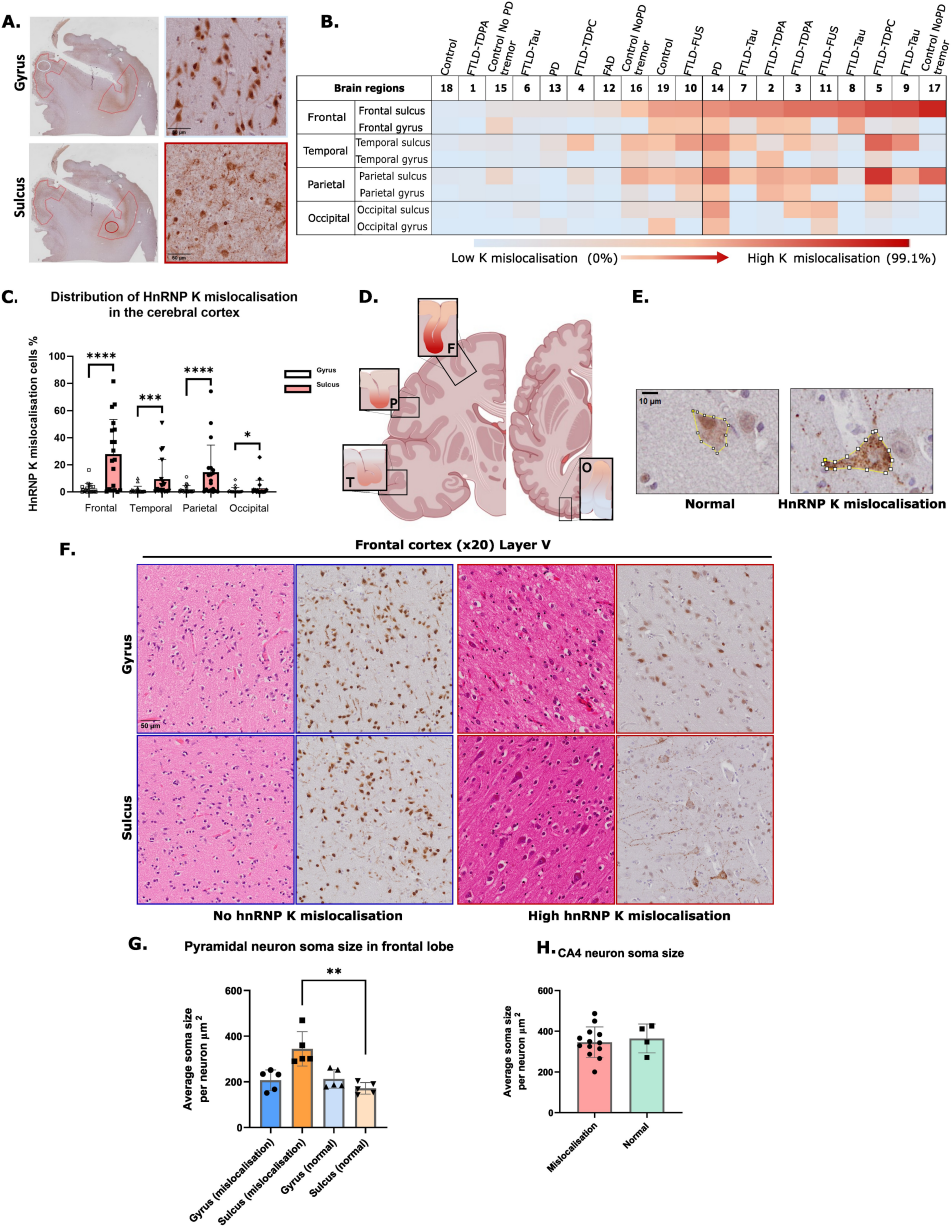
Differences in hnRNP K mislocalisation between cortical gyri and sulci. (A) Representative images of hnRNP K‐stained section highlighting differences in hnRNP K mislocalisation between the gyrus and sulcus. (B) HnRNP K mislocalisation was quantified separately in the sulcus and gyrus for each cortical region. The heat map presents data from the four brain regions and eight regions of interest (ROIs). The ordering follows the level of hnRNP K mislocalisation in the frontal sulcus, ranked from high to low, moving from left to right. Colour gradients from blue to red represent low to high levels of hnRNP K mislocalisation. (C) Distribution of hnRNP K mislocalisation in the sulcus and gyrus from the four cortical regions, frontal, temporal, parietal and occipital. In all cortical regions, the mislocalisation of hnRNP K in the sulcus is significantly higher than in the gyrus. Frontal gyrus vs. frontal sulcus: ****p < 0.0001 (Wilcoxon matched‐pairs test). Temporal gyrus vs. sulcus: ***p < 0.0005 (Wilcoxon matched‐pairs test). Parietal gyrus vs. sulcus: ****p < 0.0001 (Wilcoxon matched‐pairs test). Occipital gyrus vs. sulcus: *p = 0.0369 (Wilcoxon matched‐pairs test). Panel (D) shows representative heat maps of hnRNP K mislocalisation in the sulcus and gyrus in the four cortical regions. Panel (E) shows how the soma size was measured in the pyramidal neurons. (F) Representative H&E and DAB images of the frontal cortex, highlighting the sulcus and gyrus, sourced from the original QuPath image. (G) Quantitative comparison of soma sizes in normal and hnRNP K mislocalised pyramidal cells in the frontal cortex (control group *n* = 5 vs. hnRNP K mislocalised group *n* = 5). One‐way ANOVA was employed to compare the four sets of data. *p* = 0.0081, ** (sulcus mislocalisation vs. gyrus mislocalisation); *p* = 0.0088, ** (gyrus control vs. sulcus mislocalisation); *p* = 0.0013, ** (sulcus control vs. sulcus mislocalisation); *p* = 0.0931, not significant (sulcus control vs. gyrus control). (H) Comparison of soma sizes between normal and hnRNP K‐mislocalised CA4 cells in the dentate gyrus (control group *n* = 4 vs. hnRNP K mislocalised group *n* = 13). *t*‐test results: *p* = 0.6704 (mislocalisation vs. control, *not significant*).

To further investigate the spatial heterogeneity of hnRNP K mislocalisation within the cortical regions, we compared gyral and sulcal regions across the frontal, temporal, parietal and occipital cortices in all 19 cases (Figure 3A–D). The 19 cases were selected to represent a varying degree of hnRNP K mislocalisation in the frontal cortical sulci (Figure 3B). Quantitative analysis revealed a significantly higher proportion of pyramidal neurons with hnRNP K mislocalisation in sulcal regions compared to their adjacent gyri across all four cortical areas (Figure 3C). This differential pattern was particularly evident in layer V pyramidal neurons. However, a few individual cases deviated from this trend, such as higher mislocalisation in the frontal gyrus of one control case (Case 15) and in the temporal gyrus of a FTLD‐TDP‐43 case (Case 2). The observed sulcus‐predominant mislocalisation mirrors regional vulnerability patterns reported in other neurodegenerative conditions, such as tau pathology in chronic traumatic encephalopathy (CTE), suggesting shared underlying mechanisms influencing susceptibility in the sulci. Through the quantitative analysis of the four cortical regions, the hnRNP K mislocalisation was most severe in the frontal cortex and lowest in the occipital cortex (Figure 3C,D).

### 3.4 Soma Size Is Altered in Pyramidal Neurons With hnRNP K Mislocalisation

The process of quantification of hnRNP K mislocalisation revealed that the size of the soma of pyramidal neurons exhibiting hnRNP K mislocalisation was increased compared to cells with normal nuclear hnRNP K staining. Using manual measurements, we determined the soma size of the pyramidal neurons in the gyri and sulci of the frontal cortices of five cases with hnRNP K mislocalisation and five cases without hnRNP K mislocalisation (Table S3). The results indicate that in the cases with hnRNP K mislocalisation, the pyramidal cells in the sulcus had significantly larger soma sizes compared to cases with no mislocalisation (Figure 3E–G). Additionally, the soma size was manually measured in pyramidal cells within the CA4 region of the hippocampus. Among 16 cases with varying degrees of mislocalisation in CA4 and four cases without mislocalisation (Table S3), no significant difference in soma size was observed between the groups (Figure 3H). This suggests that the difference in soma size is restricted to cortical sulcal regions.

### 3.5 Widespread hnRNP K Mislocalisation Across Multiple Brain Regions

**FIGURE 4:**
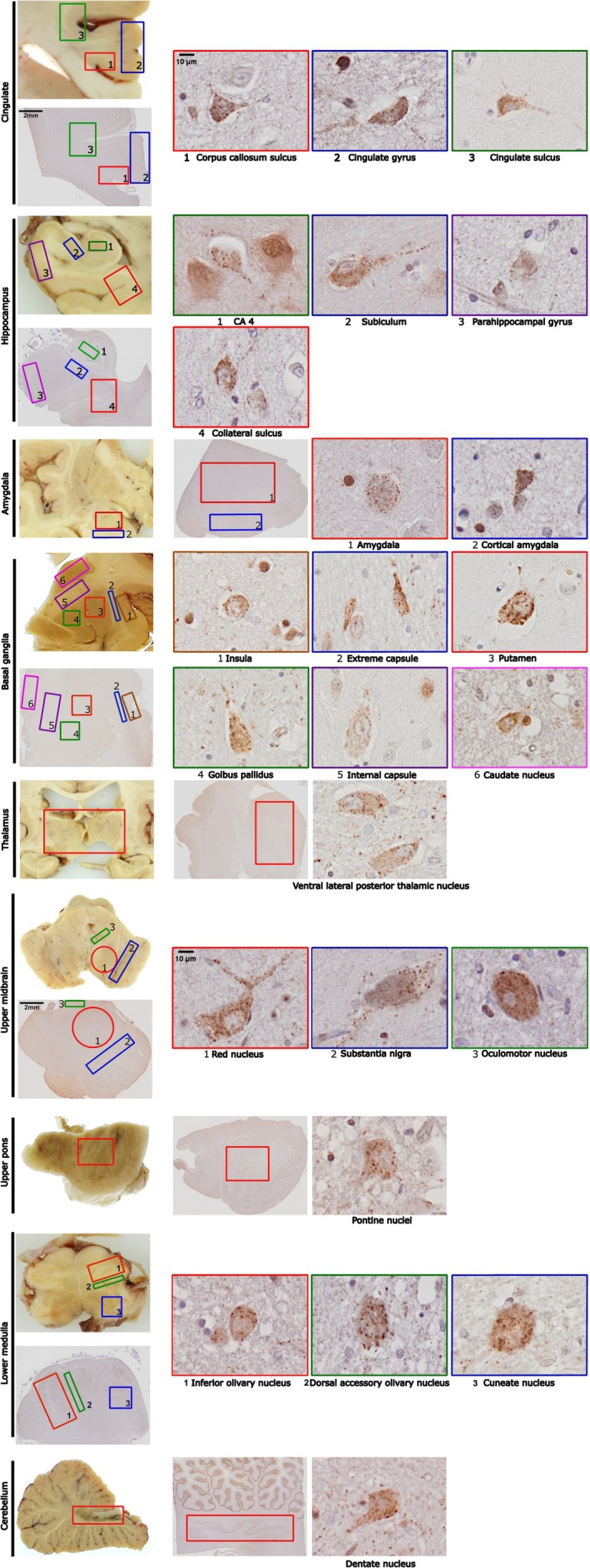
Representative images of hnRNP K mislocalisation in various cell types across different brain regions. The images show the location of hnRNP K in the neurons from nine distinct brain regions, each represented by a macro image and a coronal section alongside its corresponding hnRNP K immunohistochemistry. Different colour boxes indicate the hnRNP K mislocalisation staining patterns observed in each colour‐matched ROI. The pattern of hnRNP K mislocalisation was consistent across various cell types and brain regions, characterised by nuclear depletion and cytoplasmic puncta.

**FIGURE 5:**
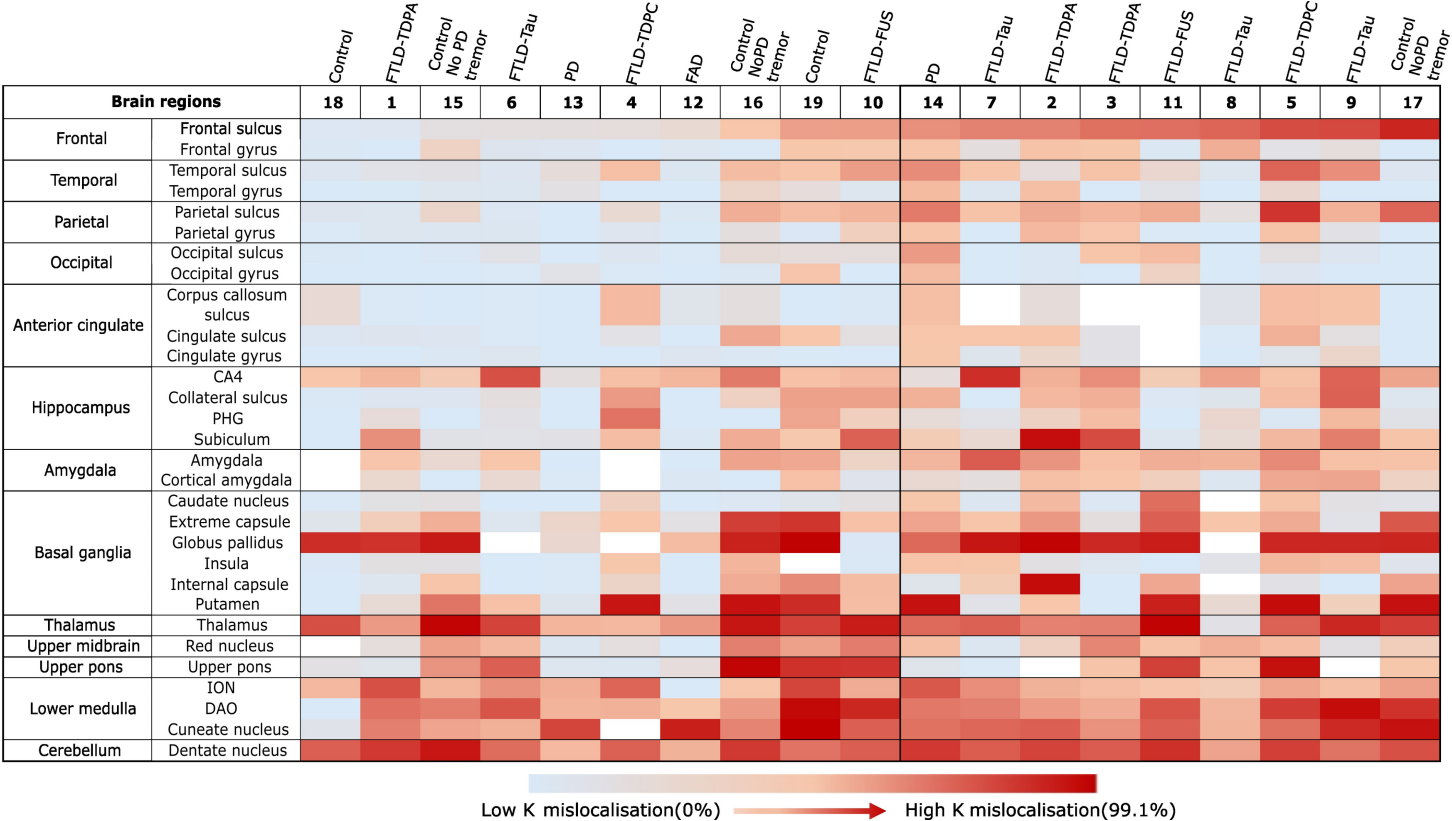
A heat map of hnRNP K mislocalisation in 13 brain regions in 19 cases. The heat map includes 30 different brain areas across 13 brain regions. Cases are sorted from low to high hnRNP K mislocalisation based on the hnRNP K mislocalisation found in the frontal sulcus and arranged left to right. Colours range from blue (low mislocalisation) to red (high mislocalisation). Blank areas indicate absence of the brain region from the case. Abbreviated in the figure as CA4 (cornu ammonis 4), PHD (parahippocampal gyrus), ION (inferior olivary nucleus) and DAO (dorsal accessory olive).

In addition to our findings in the four cortical regions, we examined a further nine different brain regions to investigate whether neurons in these brain areas also exhibited hnRNP K mislocalisation. Widespread mislocalisation of hnRNP K was observed across several anatomically and functionally distinct brain regions, suggesting that this pathological feature is not confined to the neocortex. We investigated additional brain regions that are commonly affected in neurodegenerative diseases (Figure 4) and also conducted a quantitative analysis of the percentage of mislocalised cells in all 13 brain regions across 19 cases. In the cingulate cortex, we observed mislocalisation in the gyri and sulci in a similar manner to the four main neocortical areas, with a tendency towards higher mislocalisation in the sulcus (Figure 5). In the hippocampus, strong punctate cytoplasmic hnRNP K staining was observed within the pyramidal neurons of the CA4 subfield, subiculum, parahippocampal gyrus and the collateral sulcus. The amygdala showed a mixed population of neurons with variable degrees of hnRNP K mislocalisation. Brain regions within the basal ganglia were also examined for hnRNP K mislocalisation, and neurons within the insula, extreme capsule, putamen, globus pallidus, internal capsule and caudate nucleus all exhibited mislocalisation. This pattern was also seen in the thalamus, where the majority of cases exhibited a high level of hnRNP K mislocalisation. Mislocalisation was also observed in the midbrain, pons and medulla. In the cerebellum, hnRNP K mislocalisation was observed predominantly in the dentate nucleus, whereas the Purkinje neurons showed no mislocalisation in any of the cases examined. Granule cells and interneurons in the molecular layer showed limited or no detectable cytoplasmic mislocalisation. The heat map in Figure 5 is arranged according to the percentage of hnRNP K mislocalisation in the frontal cortex sulcus, from lowest to highest (left to right). By comparing mislocalisation levels in the frontal sulcus, the heat map provides a more comprehensive visualisation of the overall distribution of hnRNP K mislocalisation across the different brain regions in the same cases and comparison across the different cases (Figure 5).

### 3.6 Regional Heterogeneity and Distribution Patterns of hnRNP K Mislocalisation Across the Brain

**FIGURE 6:**
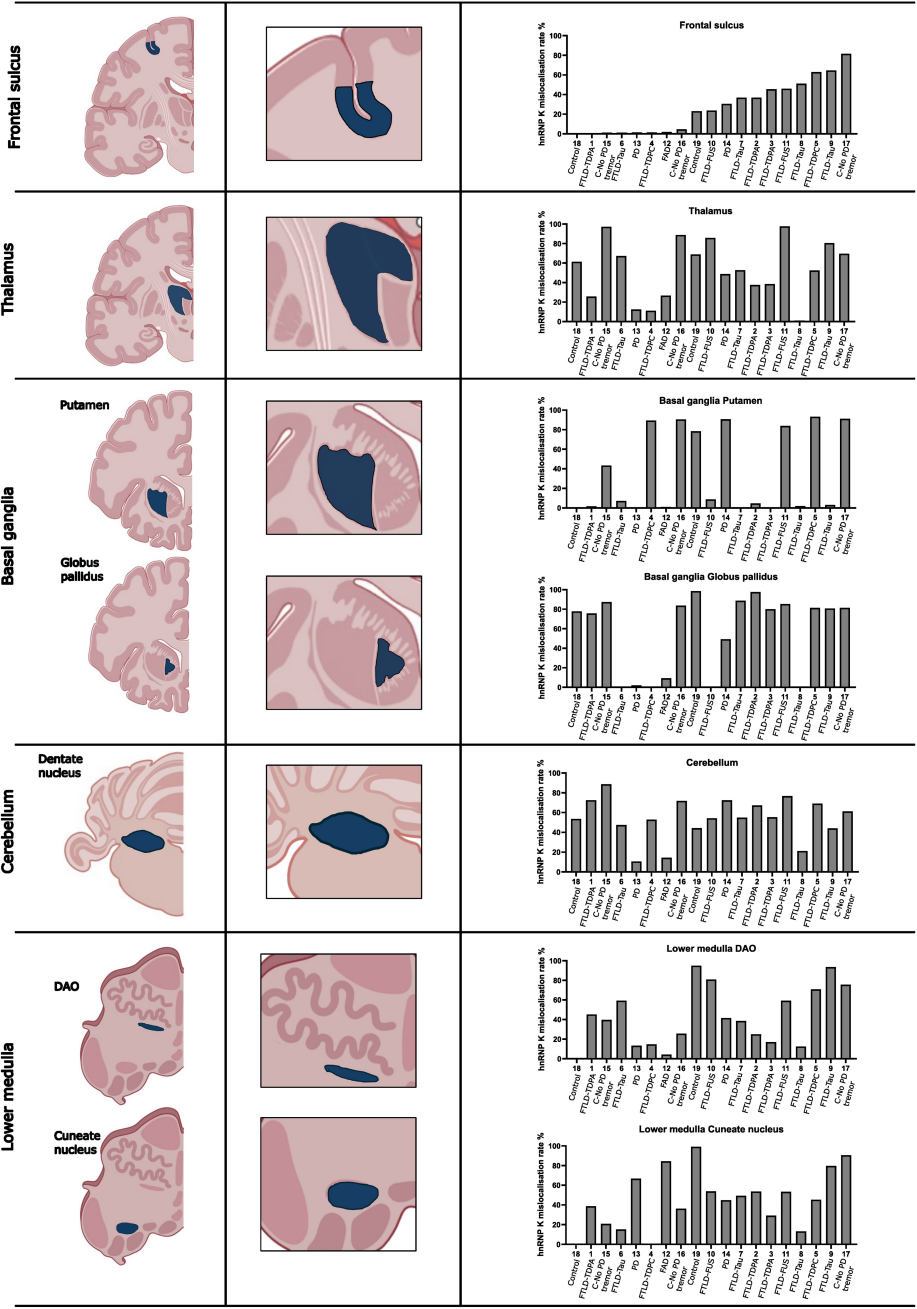
Brain regions with high levels of hnRNP K mislocalisation. Quantitative analyses of the frontal sulcus were used as standard values to compare mislocalisation levels across different brain regions. This comparison identified several regions with high hnRNP K mislocalisation, including the thalamus, basal ganglia, cerebellum and lower medulla. In the figure, the dark blue area in the first and second column highlights the location of each corresponding brain region. The final column displays the quantitative analysis results for each brain region. The numbers under the bar chart represent case numbers used in the study.

While prior work from our group has focused largely on the frontal cortex, the present analysis revealed that hnRNP K mislocalisation is not uniformly distributed across the different cases and does not correlate consistently with levels observed in the frontal lobe. In fact, contrary to our initial expectation, the frontal cortex was not the region most severely affected. Instead, several subcortical and posterior brain regions demonstrated even higher levels of hnRNP K cytoplasmic mislocalisation. To systematically assess regional variability, we ranked the 19 cases according to their hnRNP K mislocalisation levels in the frontal cortex, then compared these rankings with corresponding values across the remaining brain regions (Figure 6). This approach allowed us to determine whether mislocalisation patterns in the frontal cortex were predictive of broader mislocalisation across the brain. Notably, the data revealed considerable inter‐regional heterogeneity. Some cases with moderate frontal involvement exhibited markedly high levels of mislocalisation in posterior or deep brain structures, while others with relatively high frontal levels showed only mild involvement elsewhere.

The thalamus emerged as one of the most frequently and severely affected regions, exhibiting high hnRNP K cytoplasmic redistribution in the majority of cases. Given the thalamus's integrative role as a hub for cortico‐subcortical communication, its consistent involvement is of particular interest. Surprisingly, mislocalisation was also observed in the thalamus of several age‐matched control cases (Cases 15–19), raising the possibility that hnRNP K mislocalisation in this region may be associated with physiological ageing or preclinical pathological processes not confined to overt neurodegenerative disease.

Similarly, both the putamen and globus pallidus of the basal ganglia demonstrated striking polarisation in mislocalisation levels, with most cases showing either very high (> 90%) or negligible cytoplasmic accumulation. This all‐or‐none distribution pattern was not correlated with the pathological diagnosis of the cases, suggesting differential regional vulnerability, which may relate to underlying neuronal subtype susceptibility, metabolic demand or region‐specific stress responses. Given the central role of the basal ganglia in motor and cognitive circuits, such stark variability may have functional and clinical relevance and warrants further investigation.

Consistent hnRNP K mislocalisation was also observed in the cerebellar dentate nucleus across all examined cases, aligning with prior reports in AD [22]. This finding supports the notion that cerebellar involvement is a reliable marker of hnRNP K dysregulation. The cerebellum is increasingly recognised for its contribution to cognitive as well as motor functions, and the link between hnRNP K pathology and age‐related cerebellar dysfunction deserves systematic exploration.

Finally, mislocalisation in the lower medulla oblongata was also prominent in a subset of cases. This region, which houses autonomic and respiratory centres, is rarely examined in protein mislocalisation studies and may represent an underappreciated site of vulnerability in diseases involving RBP dysregulation.

### 3.7 Cell‐Type‐Specific Variability in HnRNP K Mislocalisation Across the Cerebellum, Hippocampus and Medulla

**FIGURE 7:**
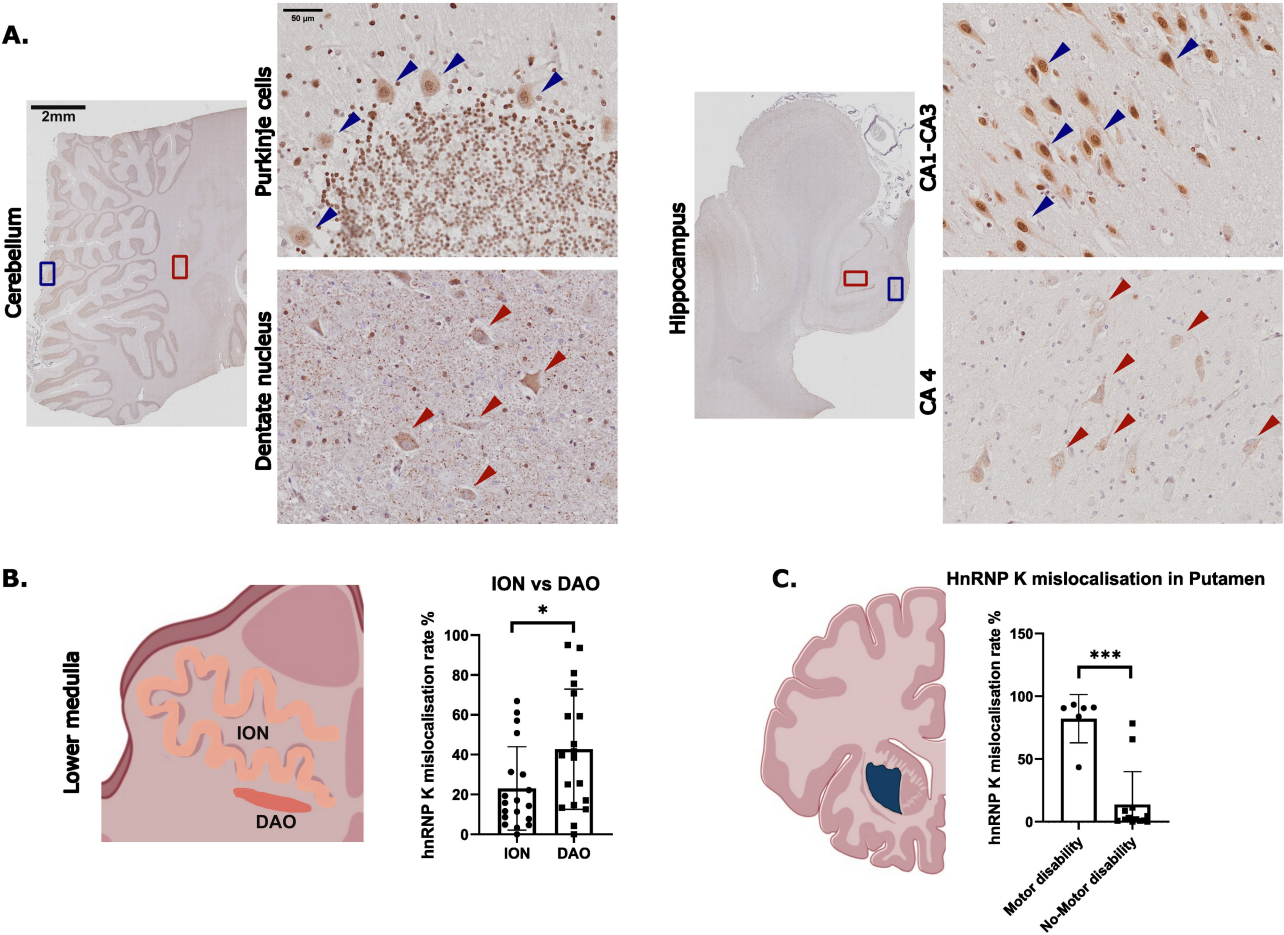
(A) Differential vulnerability of hnRNP K in cerebellum and hippocampus. Stained sections revealed region‐ and cell‐specific differences in hnRNP K mislocalisation. Purkinje cells in the cerebellum showed no mislocalisation, while the dentate nucleus exhibited extensive mislocalisation. In the hippocampus, pyramidal cells in CA1‐CA2 had no mislocalisation, CA3 showed minimal levels and CA4 and the dentate gyrus displayed high mislocalisation. These findings suggest selective vulnerability of certain neurons to hnRNP K mislocalisation. (B) HnRNP K mislocalisation in olivary cells: dorsal accessory olive (DAO) vs. inferior olivary nucleus (ION). HnRNP K mislocalisation levels were significantly higher in the DAO compared to the ION. DAO vs. ION (high mislocalisation cases): *p = 0.0230 (Wilcoxon matched‐pairs test). (C) HnRNP K mislocalisation in the Putamen: motor disability vs. no‐motor disability cases. Among the 19 recorded cases, six of them had movement disorders: Cases 15–17 and 5 with no PD tremor. Case 14 has dyskinesia, and Case 11 has facial dyskinesia. Quantitative analysis showed that hnRNP K mislocalisation levels were significantly elevated in the putamen of these cases compared to non‐dyskinetic cases. ***p = 0.0003 (Mann–Whitney test).

Variations in hnRNP K vulnerability across different cell types were observed in the cerebellum, hippocampus and medulla. Quantitative analysis of all cases revealed that Purkinje cells in the cerebellum exhibited no hnRNP K mislocalisation. However, hnRNP K mislocalisation was prevalent in the cerebellar dentate nucleus (Figure 7A). A similar pattern was observed in the hippocampus, where pyramidal cells in CA1–CA3 rarely exhibited mislocalisation, whereas mislocalisation was more frequently detected in the interconnected pyramidal‐like mossy cells of CA4 (Figure 7A).

Additionally, varying levels of hnRNP K mislocalisation were observed in the olivary nucleus of the medulla. The dorsal accessory olive (DAO) and the inferior olivary nucleus (ION) are subdivisions of the inferior olivary complex, each serving distinct roles in cerebellar processing. The DAO primarily receives input from the spinal cord and brainstem and projects to the vermis and intermediate cerebellum, supporting postural control and the coordination of gross motor movements. In contrast, the ION receives input from the cerebral cortex via the red nucleus and projects to the lateral cerebellar hemispheres, contributing to fine motor control, timing and motor learning. HnRNP K mislocalisation levels were generally higher in the DAO than in the ION. In cases exhibiting high mislocalisation in the frontal sulcus, the DAO showed significantly greater mislocalisation than the ION (Figure 7B).

### 3.8 Association Between hnRNP K Mislocalisation and Dyskinesia

In the initial analysis, three control cases (Cases 17–19) were examined. Quantitative assessment across 13 brain regions demonstrated that one control case (Case 17) showed markedly elevated hnRNP K mislocalisation in the putamen relative to the other control cases. Review of the associated clinical history identified a 19‐year history of non‐Parkinson's disease (non‐PD) tremor in this individual. In light of this finding, two additional control cases (Cases 15 and 16) with documented non‐PD tremor were subsequently analysed to further evaluate this observation. Among the 19 cases analysed in this study, eight exhibited high levels of hnRNP K mislocalisation in the putamen. Of these, five cases had documented clinical evidence of dyskinesia: three cases presented with non‐PD tremor (Cases 15–17), one case with generalised dyskinesia (Case 14) and one case with facial dyskinesia (Case 11). An additional case had limited clinical documentation, precluding assessment of motor symptoms (Case 5).

Quantitative analysis revealed significantly higher levels of hnRNP K mislocalisation in the putamen of cases with dyskinesia compared to those without motor symptoms (Figure 7C). To determine whether this association was confounded by age, we assessed the correlation between age at death and hnRNP K mislocalisation levels in the putamen. No significant relationship was observed, suggesting that the observed increase in mislocalisation in dyskinetic cases is unlikely to be age‐dependent (Figure 7C).

## 4 Discussion

In this study, we systematically examined the regional and cellular distribution of hnRNP K in post‐mortem human brain tissue from individuals with various neurodegenerative diseases and neurologically normal controls. We found that hnRNP K mislocalisation is widespread throughout the brain but exhibits distinct regional and cell‐type specific patterns, with nuclear depletion and cytoplasmic aggregation most pronounced in the frontal cortex, thalamus, basal ganglia, medulla oblongata and deep cerebellar nuclei. Importantly, this mislocalisation occurred in regions with healthy neuronal populations, suggesting that hnRNP K mislocalisation may represent an early or independent pathological event. Although previous work has established hnRNP K mislocalisation in FTLD frontal cortex [21] and in the hippocampus [22], this analysis extends these findings across 13 brain regions and different neurodegenerative diseases, highlighting its heterogeneous distribution across the brain. Mislocalisation was not limited to the neocortex but was also seen in subcortical regions integral to motor and cognitive function, including the putamen and thalamus. Interestingly, Purkinje cells in the cerebellar cortex and CA1–CA2 pyramidal neurons in the hippocampus were consistently spared, suggesting selective neuronal vulnerability, potentially underpinned by differential metabolic demands or protein quality control mechanisms [24, 25, 26].

Quantitative analysis revealed that sulcal regions of the cerebral cortex have significantly greater hnRNP K mislocalisation than gyral regions, a pattern reminiscent of the distribution of tau pathology observed in CTE cases [27]. This may reflect increased susceptibility of sulcal neurons to ischaemic stress or altered clearance mechanisms. Among affected neurons, pyramidal cells exhibited the most intense cytoplasmic accumulation, particularly in the frontal cortex, which may reflect their high metabolic and translational activity and sensitivity to dysregulation of RNA metabolism [28].

A particularly intriguing observation was that frontal pyramidal neurons with hnRNP K mislocalisation displayed enlarged soma, potentially indicative of neuronal hypertrophy. While the underlying mechanisms remain uncertain, this may relate to perturbed RNA homeostasis, cytoskeletal rearrangement or altered protein turnover, a hypothesis consistent with hnRNP K's known roles in mRNA translation, stability and transport [21, 29].

Strikingly, cases with dyskinesia showed significantly higher hnRNP K mislocalisation in the putamen, implicating the loss of hnRNP K function in motor circuit dysregulation. Although the potential contribution of concomitant proteinopathies to putaminal hnRNP K pathology in disease cases cannot be excluded, findings in the control cohort provide important context for interpretation. Notably, comparably elevated levels of hnRNP K mislocalisation were observed in the putamen of control individuals without known neurodegenerative proteinopathy but with a long‐standing history of tremor. Furthermore, significant differences in putaminal mislocalisation were identified between dyskinesia and non‐dyskinesia groups. Together, these observations suggest that hnRNP K mislocalisation in the putamen may not be exclusively driven by classical protein aggregation pathology but could instead reflect vulnerability associated with movement‐related dysfunction. This aligns with the putamen's role in movement control and suggests that RBP mislocalisation may contribute to motor symptoms in neurodegenerative disorders through disruption of synaptic or dopaminergic signalling.

Our data also revealed that hnRNP K mislocalisation does not necessarily correlate with classical neuropathological hallmarks. For example, despite prominent tau pathology in the hippocampus of AD cases, hnRNP K mislocalisation was minimal in the parahippocampal gyrus. This implies that hnRNP K aggregation may proceed via independent or parallel pathways, similar to observations with other RBPs such as TDP‐43 and FUS [30, 31]. It raises the possibility that hnRNP K mislocalisation may act as a secondary stressor or synergistic factor that exacerbates vulnerability in regions already compromised by other proteinopathies. The absence of mislocalisation in Purkinje cells and CA1–CA2 neurons suggests these cells may be protected by efficient protein degradation pathways such as the autophagy‐lysosome system or ubiquitin‐proteasome system (UPS), or through synaptic plasticity mechanisms like LTP/LTD that confer resilience [32, 33]. Conversely, the dentate gyrus and deep cerebellar nuclei, regions less frequently studied in this context, showed prominent mislocalisation, warranting further investigation into their role in hnRNP K‐related pathology.

An important consideration is whether hnRNP K mislocalisation represents a primary pathological process or, at least in part, an age‐associated phenomenon. Although the severity and regional extent of mislocalisation were consistently greater in neurodegenerative disease cases, hnRNP K redistribution was also detectable in aged neurologically normal controls. This indicates that mislocalisation is not exclusively disease‐specific. Rather, it may reflect an age‐related vulnerability of RBP homeostasis that becomes exacerbated in the context of neurodegenerative stressors. In this framework, hnRNP K mislocalisation could represent a continuum phenomenon arising at low levels during normal ageing but reaching a pathological threshold in disease, where nuclear depletion and cytoplasmic accumulation may contribute to impaired RNA metabolism, proteostasis imbalance and neuronal dysfunction. Thus, while not uniquely diagnostic of disease, the increased burden and altered regional distribution observed in neurodegenerative cases support the interpretation that hnRNP K mislocalisation is likely to have pathological significance when present at higher levels or in vulnerable neuronal populations.

Limitations of this study include the relatively small cohort size and reliance on semiquantitative image analysis, which may have missed subtle pathological changes. Additionally, due to the post‐mortem nature of the samples, we cannot determine the temporal sequence of hnRNP K redistribution or its direct causal role in neurodegeneration.

In conclusion, our study reveals a complex, regionally selective pattern of hnRNP K mislocalisation across the aged and neurodegenerative human brain, extending beyond previously described cortical regions. These findings contribute to a growing understanding of hnRNP K as part of a broader class of RNA‐binding proteinopathies and underscore the need for further mechanistic studies to elucidate its role in neurodegenerative disease pathogenesis.

Taken together, our findings demonstrate that hnRNP K mislocalisation is a widespread but non‐uniform feature of the neurodegenerative brain and the aged control brain. It appears to be modulated by neuronal subtype, anatomical location and possibly disease‐specific stressors and regional metabolic activity. Notably, hnRNP K has been implicated in regulating gene expression, myelination and synaptic plasticity—functions that are essential for maintaining neuronal health and cognitive integrity [18]. Given these critical roles, mislocalisation of hnRNP K may result in widespread cellular dysfunction.

## Author Contributions

A.G. and T.L. conceptualised and designed the experiments. X.J. generated neuropathological data. T.L., A.G., C.E.T. and X.J. interpreted the data. All authors read and approved the final version of the manuscript.

## Ethics Statement

The procurement and use of human tissues in this study were in accordance with the UK Human Tissue Act 2004. All samples were supplied, anonymised by Queen Square Brain Bank, UCL Queen Square Institute of Neurology and had full research consent (REC 23/LO/0044).

## Conflicts of Interest

The authors declare no conflicts of interest.

## Funding

T.L., A.G. and X.J. are supported by Alzheimer's Society, 579. T.L. is supported by Alzheimer's Research UK and the Association of Frontotemporal Dementia. A.G. is supported by BRAIN nonclinical fellowship and My Name'5 Doddie Foundation.

## Supporting information


**Table S1:1.** Cells in different brain regions.

**Table S1:2.** Cells in different brain regions.

**Table S2:** Quantitative percentage of images quantified of different brain regions in 19 cases. Random images are selected with intervals existing between each one to avoid duplicate areas. The maximum area from which ROI can be selected is 45%.

**Table S3:** Demographic data for cohorts used in soma size measurement. AOO stands for age of onset; AAD stands for age at death; na stands for not applicable.

**Figure S1:** Representative ROI (region of interest) of maximum images selection.

## Data Availability

The data that support the findings of this study are available from the corresponding author upon reasonable request.
