# Supplementary figures and images for: Widespread hnRNP K Mislocalisation Suggests Differential Neuronal Vulnerability in the Neurodegenerative and Ageing Human Brain
Source: Neuropathol Appl Neurobiol. 2026 Apr 8;52(2):e70072. doi: 10.1111/nan.70072 (PMC13062711; doi:10.1111/nan.70072)

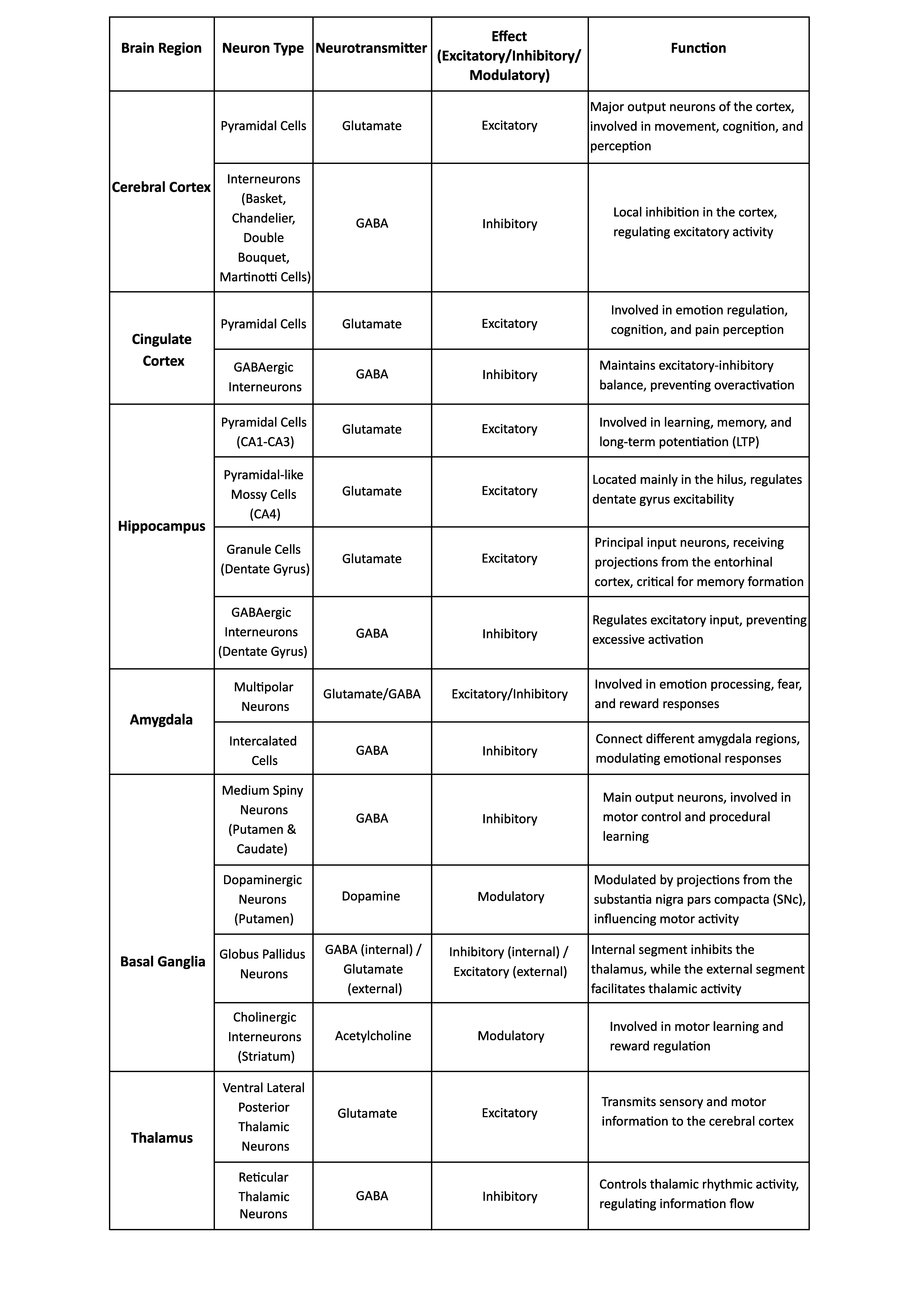

Supplement: Supplementary file 1 — Table S1:1. Cells in different brain regions. [file NAN-52-e70072-s001.jpg]

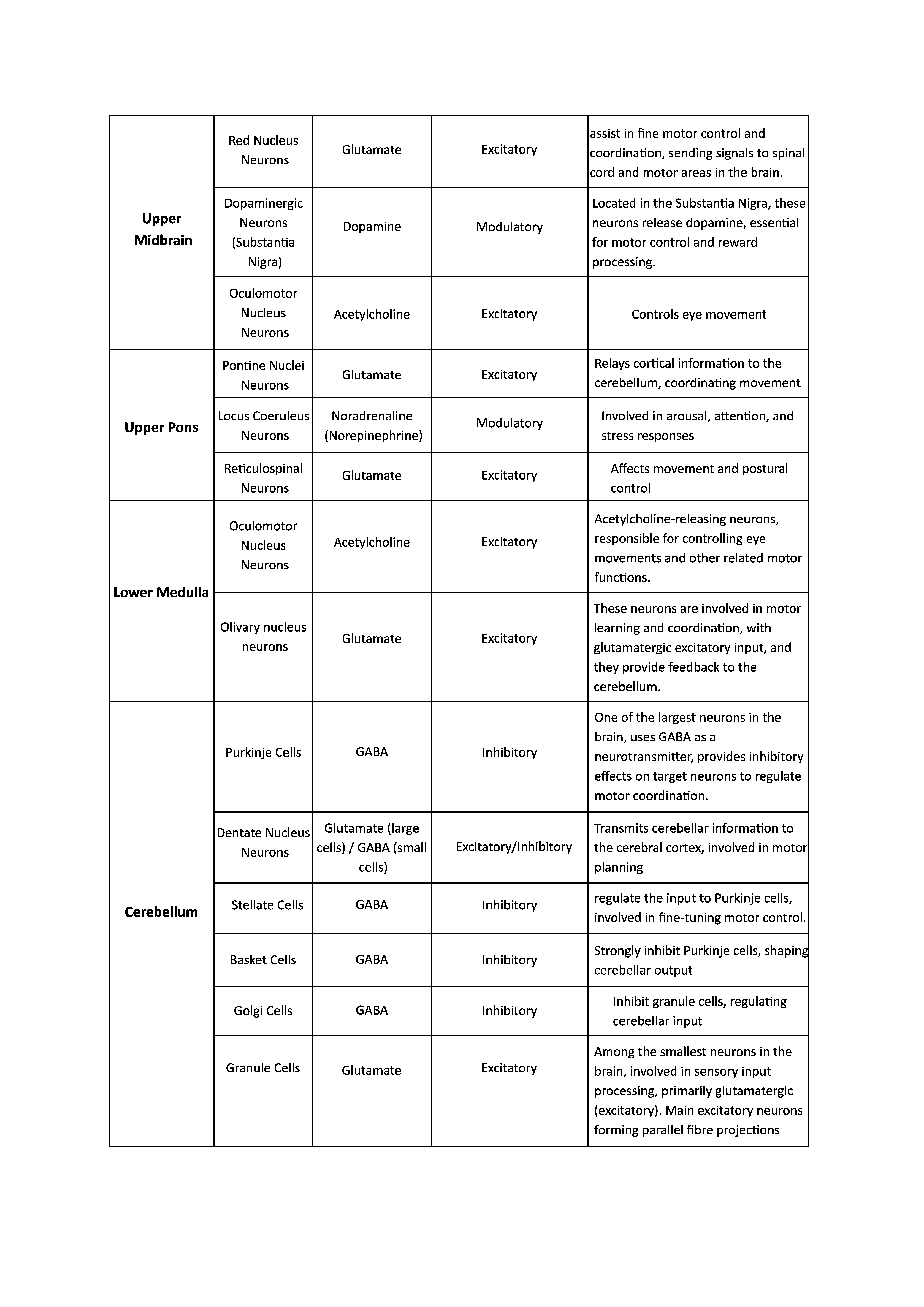

Supplement: Supplementary file 2 — Table S1:2. Cells in different brain regions. [file NAN-52-e70072-s004.jpg]

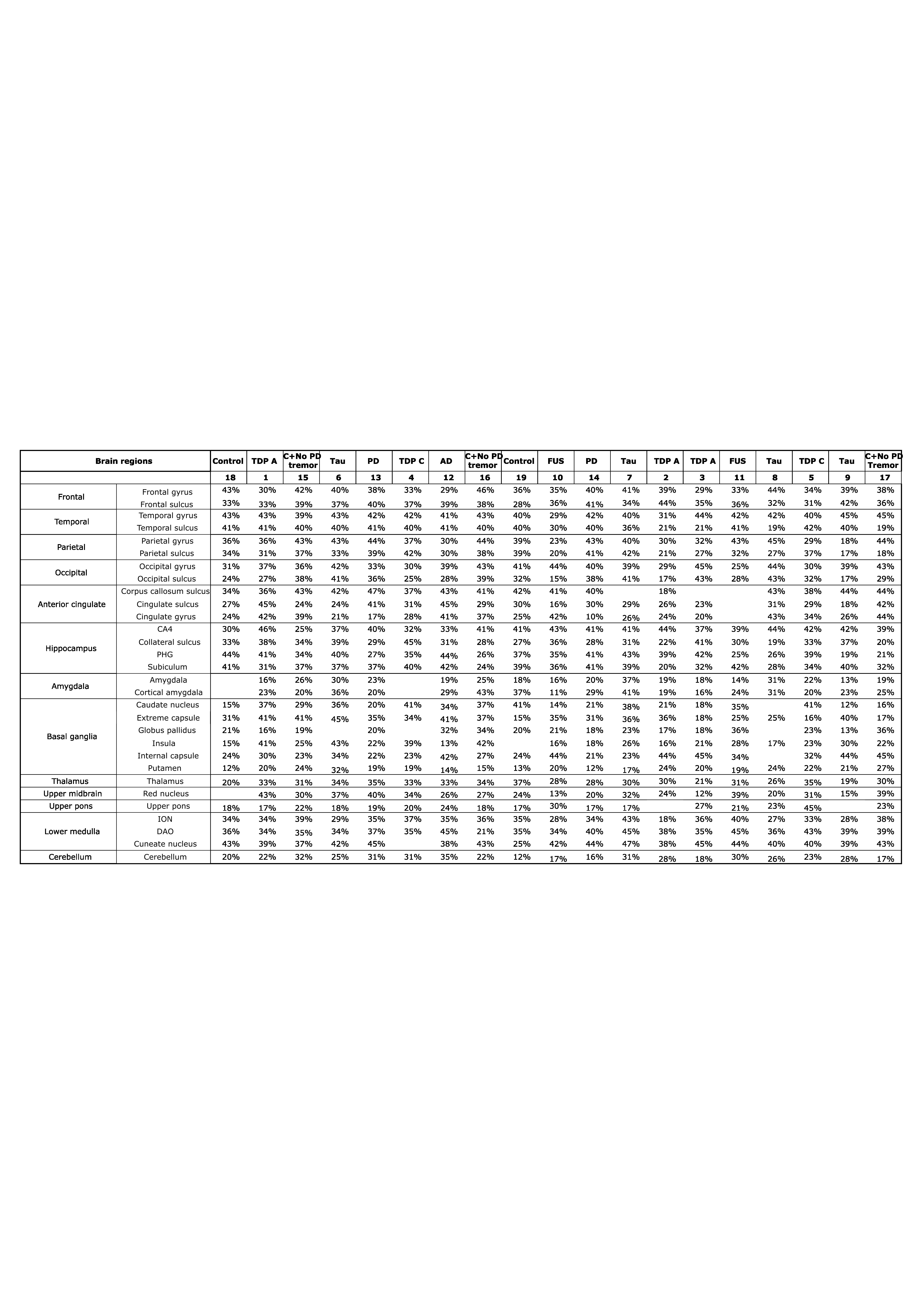

Supplement: Supplementary file 3 — Table S2: Quantitative percentage of images quantified of different brain regions in 19 cases. Random images are selected with intervals existing between each one to avoid duplicate areas. The maximum area from which ROI can be selected is 45%. [file NAN-52-e70072-s003.jpg]

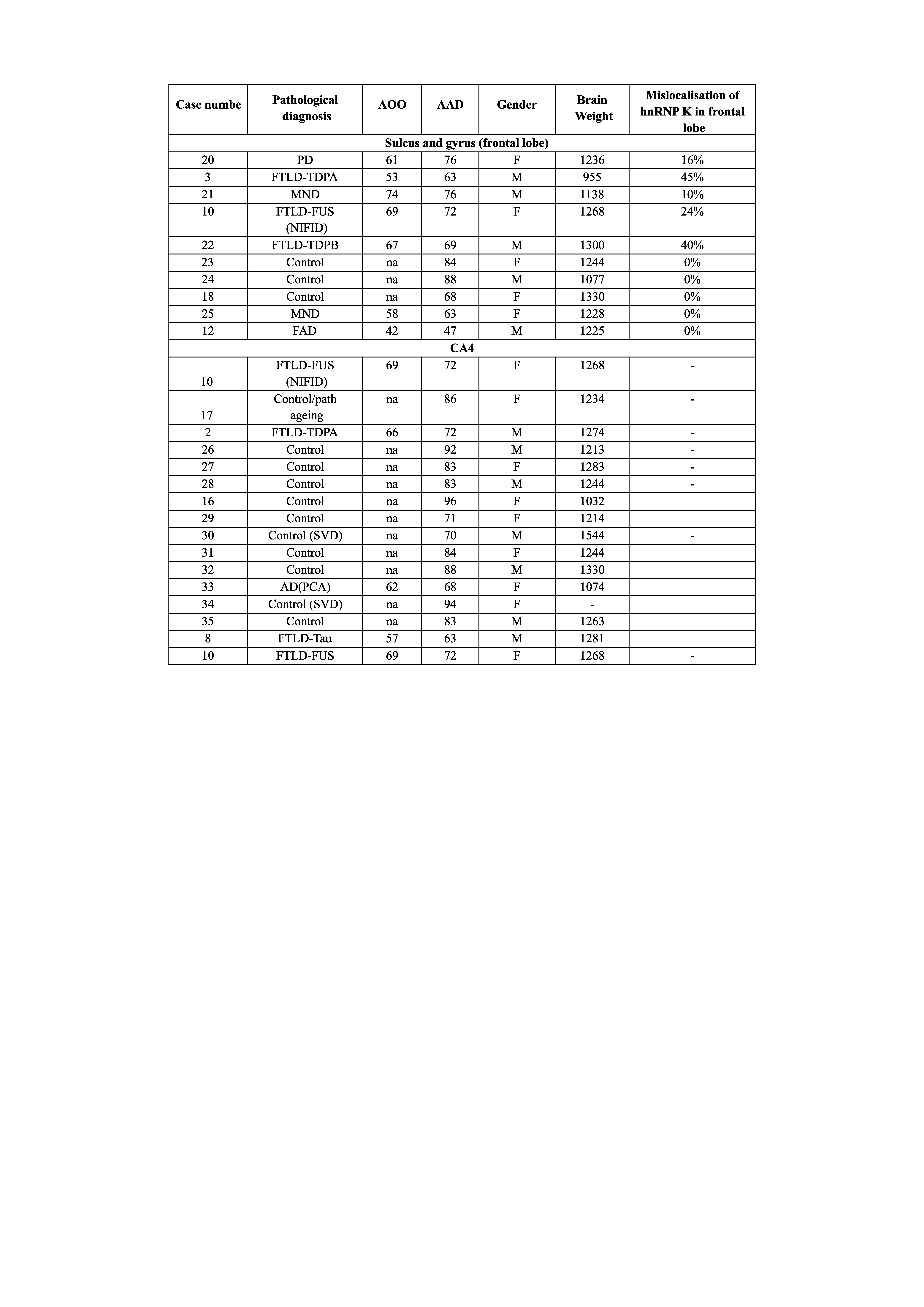

Supplement: Supplementary file 4 — Table S3: Demographic data for cohorts used in soma size measurement. AOO stands for age of onset; AAD stands for age at death; na stands for not applicable. [file NAN-52-e70072-s002.jpg]

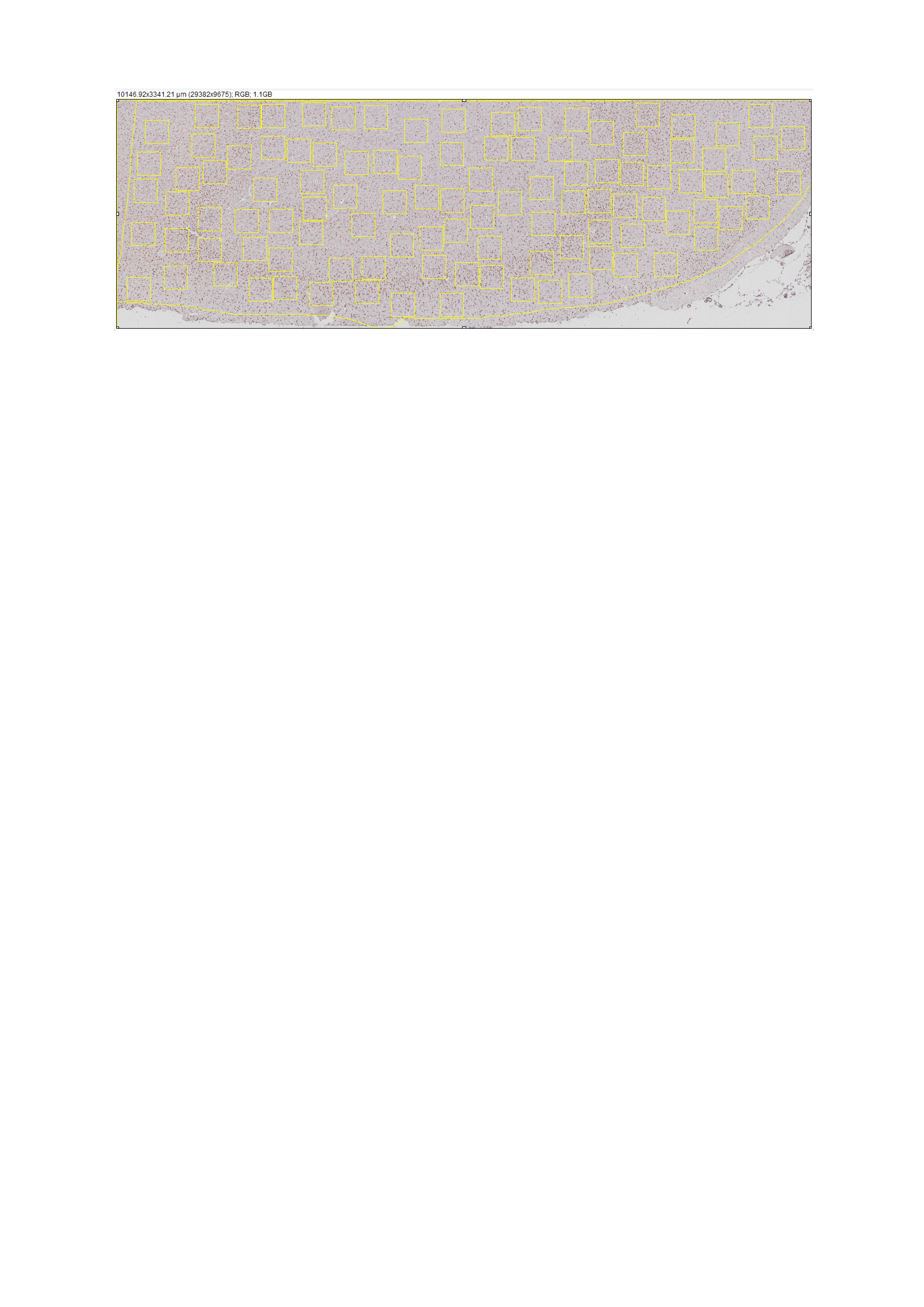

Supplement: Supplementary file 5 — Figure S1: Representative ROI (region of interest) of maximum images selection. [file NAN-52-e70072-s005.jpg]
